# Experimental design approach for deposition optimization of RF sputtered chalcogenide thin films devoted to environmental optical sensors

**DOI:** 10.1038/s41598-017-03678-w

**Published:** 2017-06-14

**Authors:** E. Baudet, M. Sergent, P. Němec, C. Cardinaud, E. Rinnert, K. Michel, L. Jouany, B. Bureau, V. Nazabal

**Affiliations:** 1 Institut des Sciences Chimiques de Rennes, UMR-CNRS 6226, Université de Rennes 1, 35042 Rennes, France; 2 Aix Marseille Université, LISA EA4672, Campus scientifique de Saint Jérôme, 13397 Marseille, France; 3 Department of Graphic Arts and Photophysics, Faculty of Chemical Technology, University of Pardubice, Studentska 573, 53210 Pardubice, Czech Republic; 4 Institut des matériaux Jean Rouxel (IMN) UMR 6502, Université de Nantes, CNRS, 44322 Nantes Cedex 3, France; 5 IFREMER, Laboratoire Détection, Capteurs et Mesures, Dpt. Recherches et Développements Technologiques, 29280 Plouzané, France; 6 BRGM, Direction Eau, Environnement et Ecotechnologies, Unité Bio-Géochimie environnementale et qualité de l’Eau, 45060 Orléans, France

## Abstract

The development of the optical bio-chemical sensing technology is an extremely important scientific and technological issue for diagnosis and monitoring of diseases, control of industrial processes, environmental detection of air and water pollutants. Owing to their distinctive features, chalcogenide amorphous thin films represent a keystone in the manufacture of middle infrared integrated optical devices for a sensitive detection of biological or environmental variations. Since the chalcogenide thin films characteristics, i.e. stoichiometric conformity, structure, roughness or optical properties can be affected by the growth process, the choice and control of the deposition method is crucial. An approach based on the experimental design is undoubtedly a way to be explored allowing fast optimization of chalcogenide film deposition by means of radio frequency sputtering process. Argon (Ar) pressure, working power and deposition time were selected as potentially the most influential factors among all possible. The experimental design analysis confirms the great influence of the Ar pressure on studied responses: chemical composition, refractive index in near-IR (1.55 µm) and middle infrared (6.3 and 7.7 µm), band-gap energy, deposition rate and surface roughness. Depending on the intended application and therefore desired thin film characteristics, mappings of the experimental design meaningfully help to select suitable deposition parameters.

## Introduction

The mid-infrared (mid-IR) spectral range is a key region for a large number of applications in diverse areas such as biology and medicine, molecular spectroscopy, ground based and space borne environmental monitoring but also an important issue of instrumentation for astronomy and astrophysics to achieve complex but very reliable instruments^1–6^. Most infrared signatures or “fingerprints” (fundamental transitions) of organic species and biomolecules are essentially located in this spectral window^4^. Thus, the infrared spectroscopy is a powerful tool for detecting and determining the composition of complex samples; it is a simple, reliable, fast, cost-efficient and non-destructive method. The development of an optical bio-chemical sensing technology for measurement in real-time is an extremely important scientific and technological issue for the diagnosis and monitoring of diseases, drug discovery, proteomics, industrial process control, environmental detection of pollutants or biological agents. Such sensors require a high stability, a high selectivity to interfering molecules and a large detection range to their improve performance. These optical sensors can be based on the existence of the evanescent field which is a fraction of the guided light that is outside the waveguide and thus can probe the external medium surrounding the waveguide at about a few hundred nanometers. That implies, considering the intrinsic characteristics of the optical sensors, the evanescent field is sensitive to the changes induced by the analyte on the sensor surface such as scattering, fluorescence and notably absorption. Then, it is crucial to be able to work at the extension toward IR waveguides, where chalcogenide glasses provide promising properties. Chalcogenide glasses provide specific optical characteristics (broad mid-IR transparency window, low phonon energies, large linear and nonlinear index of refraction) which make them appropriate to be used in integrated optical devices for a sensitive detection of biological or environmental variations^4, 7–11^. Amorphous chalcogenides allow light control at very small scales and are suitable for high index contrast photonic devices, with a compact design requiring a small space and can also be deposited on various substrates^12–14^. The fabrication of chalcogenide thin films for developing integrated optical devices is usually carried out using chemical vapor deposition^15^, thermal evaporation^16–18^, pulsed laser deposition (PLD)^19–23^, or radio frequency (RF) sputtering^14, 24–26^.

Since the thin film characteristics, i.e. stoichiometric conformity, morphology, topography, structure, or optical properties can be affected by the deposition process; the choice of the deposition technique is decisive. Based on our own experience comparing different physical deposition techniques like PLD, evaporation or RF sputtering^27–30^ and recently published results^31–33^, it appears that the RF sputtering is a method of choice for photonics development. Consequently, in this study the RF magnetron sputtering was chosen for chalcogenide layer deposition devoted to mid-IR sensor due to the aptitude of a quite good adherence, high composition control, good homogeneity and uniformity and industrial process transfer prospect. However, as mentioned in the literature^31, 34^, the sputtering deposition parameters must be extremely well controlled to prevent a chalcogenide film growth with an unsuitable morphology and topography that can dramatically increase the optical losses of chalcogenide waveguide or can considerably modify the film surface which could affect their functionalization required for a selective and sensitive detection^4, 11, 35^.

Indeed, to develop an optical integrated system for mid-IR sensors which requires precise opto-geometric characteristics of the chalcogenide layers^10^, it is essential to adequately determine the influence of deposition parameters. By the role discrimination of each factor and their possible correlation, it will be possible to control thin film characteristics such as thickness, refractive index, roughness, or chemical composition. The approach based on the experimental design is undoubtedly a way to be explored allowing fast optimization of chalcogenide glasses deposition by means of Physical Vapor Deposition (PVD) processes that are used for a thin films growth onto the appropriate substrate.

In the ternary Ge-Sb-Se system, pseudo-binary (GeSe_2_)_100−x_(Sb_2_Se_3_)_x_ (x = 5, 10, 20, 30, 40, 50 and 60) glasses were already studied for photonics applications concerning nonlinear optics or sensors^10, 11, 35–40^. For this study, two nominal compositions, Ge_28.1_Sb_6.3_Se_65.6_ (x = 10) and Ge_12.5_Sb_25_Se_62.5_ (x = 50), were selected for their excellent mid-IR transparency, high stability against crystallization and refractive index contrast allowing optical waveguiding in mid-IR^10, 11, 39^. The fabrication of these two different chalcogenide films has been optimized by the experimental design approach in order to fully meet the criteria for producing optical components devoted to optical bio-chemical sensor applications, for which controlling the layer homogeneity is fundamental for thicknesses of few µm (from 1 to 6 µm).

The experimental design approach was proposed to quantify the relationship between the different thin film characteristics and the input variables while minimizing the number of experiments. The experimental design was defined considering potentially the most influential factors that are worthy to be studied. To do this, preliminary experiments were performed to assess importance of individual factors and to fix the most important ones for the experimental design analysis. The aim of this work is to reveal significant experiments among their large set to determine a connection between deposition parameters and thin films characteristics^35, 38^. Thus, this study shows interactions between deposition parameters and determines the main deposition parameters influencing selenide thin films properties. Three input variables or factors have been selected for their expected significant influence: argon pressure, working power and deposition time (Table 1). Six different responses necessary for the optical waveguide development have been consequently investigated: deposition rate, chemical composition, refractive indices (*n*) in near-IR and mid-IR, band-gap energy, and surface roughness. The knowledge of the deposition parameters influence on the mentioned responses will consequently allow achieving accurate manufacturing of the optical waveguide proposed for the mid-IR environmental sensor working in the range of 3–12 µm.

## Results and Discussion

In order to determine the optimal conditions for the film deposition parameters, an experimental design study was carried out. This approach proposes to realize the most significant experiments to obtain the maximum information on the characteristics of the thin films obtained from Ge_28.1_Sb_6.3_Se_65.6_ and Ge_12.5_Sb_25_Se_62.5_ glass targets.

For this study, response surface methodology (RSM) was proposed to evaluate the influence of the factors and to optimize the deposition parameters. In RSM, an empirical mathematical model is postulated to express the response as a function of studied parameters and then to predict responses in the whole domain of interest. In this case, the second order polynomial model was postulated to capture the possible nonlinear effects and curvature in the domain (Equation 1):

1 $$Y={b}_{0}+{b}_{1}{X}_{1}+{b}_{2}{X}_{2}+{b}_{3}{X}_{3}+{b}_{11}{X}_{1}^{2}+{b}_{22}{X}_{2}^{2}+{b}_{33}{X}_{3}^{2}+{b}_{12}{X}_{1}{X}_{2}+{b}_{13}{X}_{1}{X}_{3}+{b}_{23}{X}_{2}{X}_{3}$$

where *Y* is the response, *X*_*i*_ are the dimensionless variables, *b*_*i*_ are the model coefficients. To estimate at best the coefficients of this model, different designs of experiments are possible and an uniform shell Doehlert^41^ design was chosen. This design consists of thirteen distinct experiments; the center point was replicated four times (n° 13 to 16, Table 2) to evaluate the repeatability of the data and to calculate the variance of the experimental error. The Doehlert design is considered as an asymmetrical design, giving different number of levels to the factors: five, seven and three values. The three key factors (X_1 _ is Ar pressure, X_2_ is working power and X_3 _ is deposition time) with their respective variation domain for the Ge_28.1_Sb_6.3_Se_65.6_ and Ge_12.5_Sb_25_Se_62.5_ thin films deposition are detailed in Table 1. The Ar working pressure ranged from 5 × 10^−2^ to 5 × 10^−3^ mbar. The sputtering power varied between 10 and 25 W for the Ge_28.1_Sb_6.3_Se_65.6_ target and between 10 and 20 W for the Ge_12.5_Sb_25_Se_62.5_ target. Note that the working pressure range and the working power were selected considering preliminary experiments of both films’ depositions. Non-selected factors less influential, like the gas flow, were fixed. Required factors for mid-IR sensor development as the nature of the substrate and the target-to-substrate distance were also settled by preliminary experiments. In case of the Ge_12.5_Sb_25_Se_62.5_ glass, the sputtering power is limited as this target is more fragile than the other one. The deposition time varied from 30 to 160 minutes. Considering these factors, the sixteen experimental conditions corresponding to the Doehlert design (Table 2) were performed for each of two above mentioned compositions.

**Table 1:** Domain of the experimental study of Ge_28.1_Sb_6.3_Se_65.6_ and Ge_12.5_Sb_25_Se_62.5_ thin films.

Factors	Number of levels	Values
Ar pressure (mbar)	5	5.10^−2^, 2.8.10^−2^, 1.6.10^−2^, 8.9.10^−3^, 5.10^−3^
Working power (W)		
Ge_28.1_Sb_6.3_Se_65.6_	7	10, 12, 15, 17, 20, 22, 25
Ge_12.5_Sb_25_Se_62.5_	7	10, 12, 13, 15, 16, 18, 20
Deposition time (min)	3	30, 95, 160

**Table 2:** Experimental parameters of the Ge_28.1_Sb_6.3_Se_65.6_ and Ge_12.5_Sb_25_Se_62.5_ thin films deposition (Ar pressure, deposition time, working power).

n°	Ar pressure (mbar)	Deposition time (min)	Working power (W)	
			Ge_28.1_Sb_6.3_Se_65.6_	Ge_12.5_Sb_25_Se_62.5_
1	5.10^−2^	95	17	15
2	5.10^−3^	95	17	15
3	2.8.10^−2^	95	25	20
4	8.9.10^−3^	95	10	10
5	2.8.10^−2^	95	10	10
6	8.9.10^−3^	95	25	20
7	2.8.10^−2^	160	20	16
8	8.9.10^−3^	30	15	13
9	2.8.10^−2^	30	15	13
10	1.6.10^−2^	30	22	18
11	8.9.10^−3^	160	20	16
12	1.6.10^−2^	160	12	12
13	1.6.10^−2^	95	17	15
14	1.6.10^−2^	95	17	15
15	1.6.10^−2^	95	17	15
16	1.6.10^−2^	95	17	15

The Y responses were selected considering their significance for the development of the environmental optical sensor. First of all, the film composition depending on the deposition parameters must be well identified. The chemical composition can have influence on the wettability of the film surface where an apolar and hydrophobic behavior must be favored, which can be advantageous for the detection of pollutant molecules. The optical properties of the film must be perfectly characterized and controlled in order to manufacture the required optical waveguide. It is especially relevant for a refractive index in the mid-IR where the absorption of the molecules is used for a bio-chemical detection. Moreover, the quality of the waveguide in term of optical losses, for instance, will depend on the roughness or the film. Thus, the dependence of these Y responses upon the different X_i_ factors, particularly the deposition time for the two latter cases, should be investigated. From the results for both compositions (Tables 3 and 4), the coefficients of the respective models were estimated by the least square method with Nemrodw software^42^. After validation, the different models were used to graphically represent the response surfaces in the domain of interest (iso-response curves); these response surfaces were used to interpret the results.

**Table 3:** Experimental results for the Ge_28.1_Sb_6.3_Se_65.6_ thin films (chemical composition, refractive index, band-gap energy and surface roughness).

n°	Chemical composition (%)			Refractive index			Band-gap energy (eV)	Deposition rate (nm/min)	Surface roughness (nm)
	ΔGe	ΔSb	ΔSe	1.55 µm	6.3 µm	7.8 µm	ΔE		
	Ge_28.8_Sb_5.8_Se_65.4_ target								
1	−0.9	−0.1	1.0	2.41	2.38	2.38	−0.13	8	2.72
2	3.5	0.5	−4.0	2.56	2.52	2.52	−0.23	23	0.37
3	0.8	1.4	−2.2	2.48	2.45	2.45	−0.14	25	4.41
4	2.1	−0.4	−1.7	2.53	2.49	2.49	−0.15	9	0.90
5	−1.3	−0.7	2.0	2.44	2.41	2.41	−0.08	5	1.77
6	2.3	0.9	−3.2	2.53	2.49	2.49	−0.15	38	0.96
7	0.3	1.0	−1.3	2.46	2.43	2.43	−0.11	17	6.46
8	0.9	−0.8	−0.1	2.54	2.50	2.50	−0.17	18	0.57
9	−2.5	−1.1	3.6	2.44	2.42	2.42	−0.10	10	2.08
10	0.1	0.2	−0.3	2.50	2.46	2.46	−0.10	25	2.39
11	2.7	0.7	−3.4	2.54	2.50	2.50	−0.15	28	0.44
12	1.5	0.4	−1.9	2.50	2.47	2.47	−0.13	10	2.78
13	1.7	0.6	−2.3	2.50	2.47	2.47	−0.14	18	1.39
14	1.9	0.5	−2.4	2.50	2.47	2.47	−0.13	19	2.59
15	1.2	0.6	−1.8	2.51	2.47	2.47	−0.15	18	2.33
16	1.4	0.6	−2.0	2.50	2.47	2.47	−0.12	18	2.72

**Table 4:** Experimental results for the Ge_12.5_Sb_25_Se_62.5_ thin films (chemical composition, refractive index, band-gap energy and surface roughness).

n°	Chemical composition (%)			Refractive index			Band-gap energy (eV)	Deposition rate (nm/min)	Surface roughness (nm)
	ΔGe	ΔSb	ΔSe	1.55 µm	6.3 µm	7.8 µm	ΔE		
	Ge_12.6_Sb_24.5_Se_62.9_ target								
1	0.7	1.1	−1.8	2.83	2.75	2.75	−0.07	10	3.56
2	3.5	1.0	−4.5	2.93	2.86	2.86	−0.15	23	0.95
3	0.7	1.4	−2.1	2.86	2.82	2.80	−0.12	24	5.24
4	3.0	0.2	−3.2	2.87	2.81	2.81	−0.07	11	0.78
5	1.1	1.4	−2.5	2.85	2.79	2.79	0.00	7	6.26
6	2.3	0.7	−3.0	2.89	2.83	2.83	−0.05	33	0.45
7	0.8	1.2	−2.0	2.84	2.79	2.79	−0.14	17	5.32
8	2.9	0.1	−3.0	2.87	2.81	2.81	−0.04	18	0.62
9	1.1	0.6	−1.7	2.86	2.81	2.80	0.03	11	0.75
10	2.0	0.6	−2.6	2.87	2.81	2.81	−0.01	27	0.99
11	2.7	0.4	−3.1	2.89	2.83	2.83	−0.07	25	0.48
12	2.0	0.0	−2.0	2.85	2.80	2.80	0.00	14	1.00
13	1.7	1.2	−2.9	2.87	2.82	2.82	−0.01	20	0.84
14	1.8	0.8	−2.6	2.87	2.81	2.81	0.00	20	0.98
15	1.9	0.6	−2.5	2.87	2.81	2.81	−0.03	20	1.02
16	1.8	1.0	−2.8	2.87	2.82	2.82	−0.02	20	0.82

### Chemical composition

The chemical composition of the bulk glass targets estimated by energy-dispersive X-ray spectroscopy (EDS) was found to be Ge_28.8_Sb_5.8_Se_65.4_ and Ge_12.6_Sb_24.5_Se_62.9_ in perfect agreement with the theoretical Ge_28.1_Sb_6.3_Se_65.6_ and Ge_12.5_Sb_25_Se_62.5_ target composition with measurement uncertainty of about ±0.5%. For the sake of simplicity, the sputtered chalcogenides films will be named in a general way by considering the theoretical compositions of the two targets; i.e. Ge_28.1_Sb_6.3_Se_65.6_ and Ge_12.5_Sb_25_Se_62.5_ thin films. The chemical compositions of the sputtered films also estimated by EDS are presented in Tables 3 and 4 showing generally that they are relatively close to the nominal composition of the bulk target depending on the selected factors. To evaluate more precisely the influence of the Ar pressure, the deposition time and the working power on the thin film composition, the variance (ΔGe, ΔSb and ΔSe) of the chemical composition of the thin films compared to the real composition of the bulk glass targets were analyzed.

#### Ge_28.1_Sb_6.3_Se_65.6_ thin films

**Figure 1:**
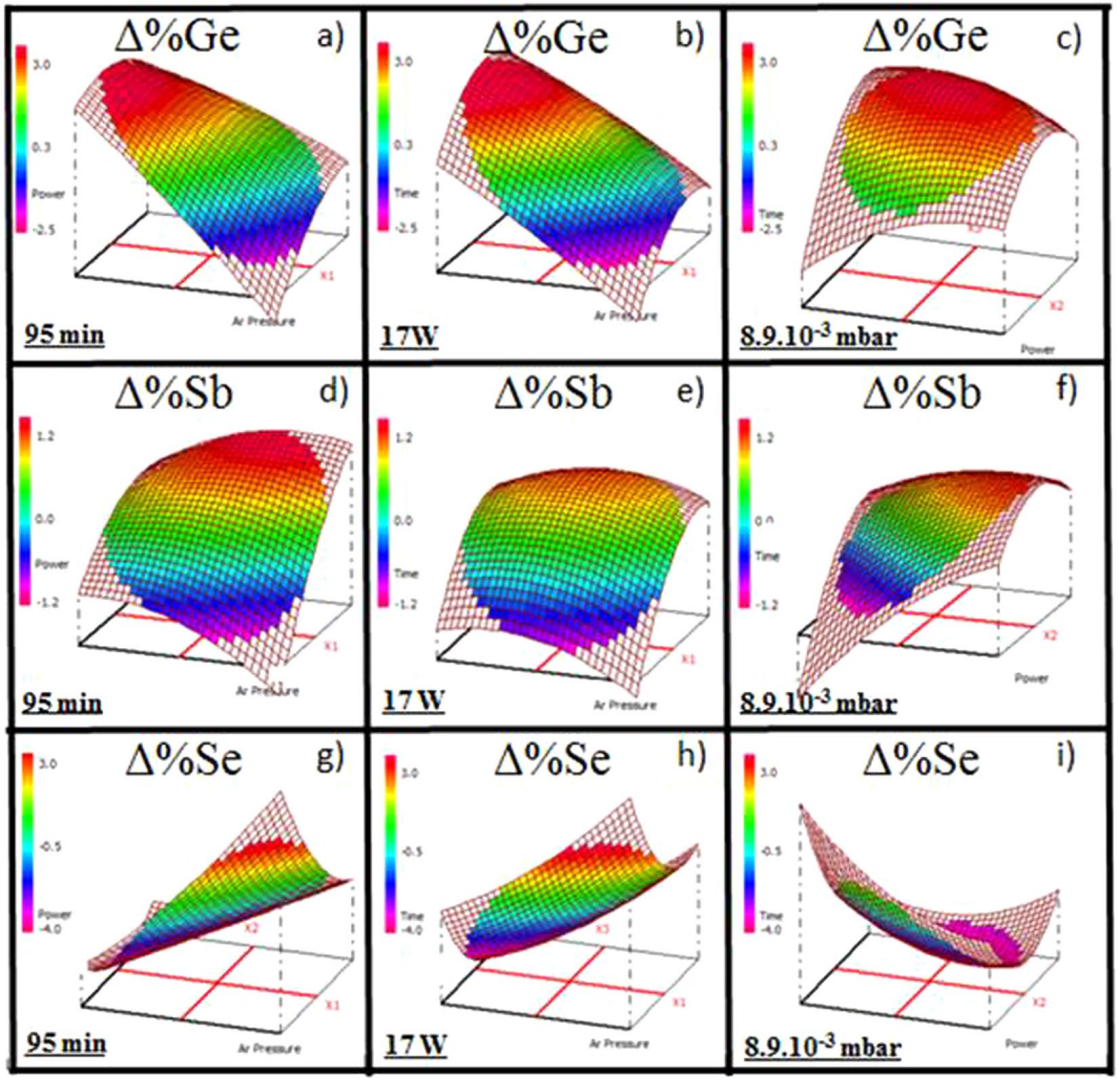
Variation of the chemical composition of the Ge_28.1_Sb_6.3_Se_65.6_ thin films for a fixed deposition time 95 min (**a**) Δ%Ge, (**d**) Δ%Sb, (**g**) Δ%Se, for a fixed working power 17 W (**b**) Δ%Ge, (**e**) Δ%Sb, (**h**) Δ%Se, for a fixed Ar pressure 8.9.10^−3^ mbar (**c**) Δ%Ge, (**f**) Δ%Sb, (**i**) Δ%Se.

Figure 1 shows the chemical composition changes of the films compared to the target (ΔC = C_film_(%) − C_target_(%)). Considering germanium, ΔGe varies from −2.5 to 3.5%. The films can have a deficit or an excess of germanium compared to stoichiometry but the films present mostly an excess of Ge (average value of ΔGe = 1%, deviation 0.3% in Table 3). Nevertheless, some deposition parameters can lead to a deficiency in germanium content (Fig. 1). In that case for a high Ar pressure and a low to moderate power, a significant decrease of the response is effectively observed. Thus, the continuous variations of ΔGe can be controlled mainly by varying the pressure of Ar. For a relative high Ar pressure and an intermediate RF power, the thin films composition in terms of its Ge content can reach the composition of the target (ΔGe ~ 0). To a lesser extent at intermediate pressure, for a short deposition time and a weak working power, ΔGe also tends to zero.

The optical waveguiding requires selenide layers with few µm thicknesses^10, 11^, consequently a long deposition time and/or a high deposition rate are required. Thus, it is quite important to control that any compositional drift during the deposition will be observed. For a fixed Ar pressure (8.9.10^−3^ mbar) and an extended deposition time, homogeneity of the response ΔGe was found constant (Fig. 1c).

In case of selenium, ΔSe values vary from −4.0 to 3.6%. We observe mainly a deficiency in selenium content (average value of −1.3%, deviation 0.3% in Table 3). Nonetheless, for a high Ar pressure associated to a low-moderate working power, ΔSe increases and Se can be in excess. As for ΔGe data, homogeneity of ΔSe composition is observed for a long deposition time (at 8.9 10^−3^ mbar) even if the power is fluctuating, ΔSe ≈ −3.3% (Fig. 1i).

A smaller variation of antimony is noted, with ΔSb from −1.1 to 1.4% (average value of 0.3%, deviation 0.1% in Table 3). In this specific case, the predominant factors are a working power and a deposition time. In detail, the response decreases for a low working power and a short deposition time. ΔSb is stable while the pressure is varying for a long deposition time and for a fixed working power (17 W) (Fig. 1e).

#### Ge_12.5_Sb_25_Se_62.5_ thin films

**Figure 2:**
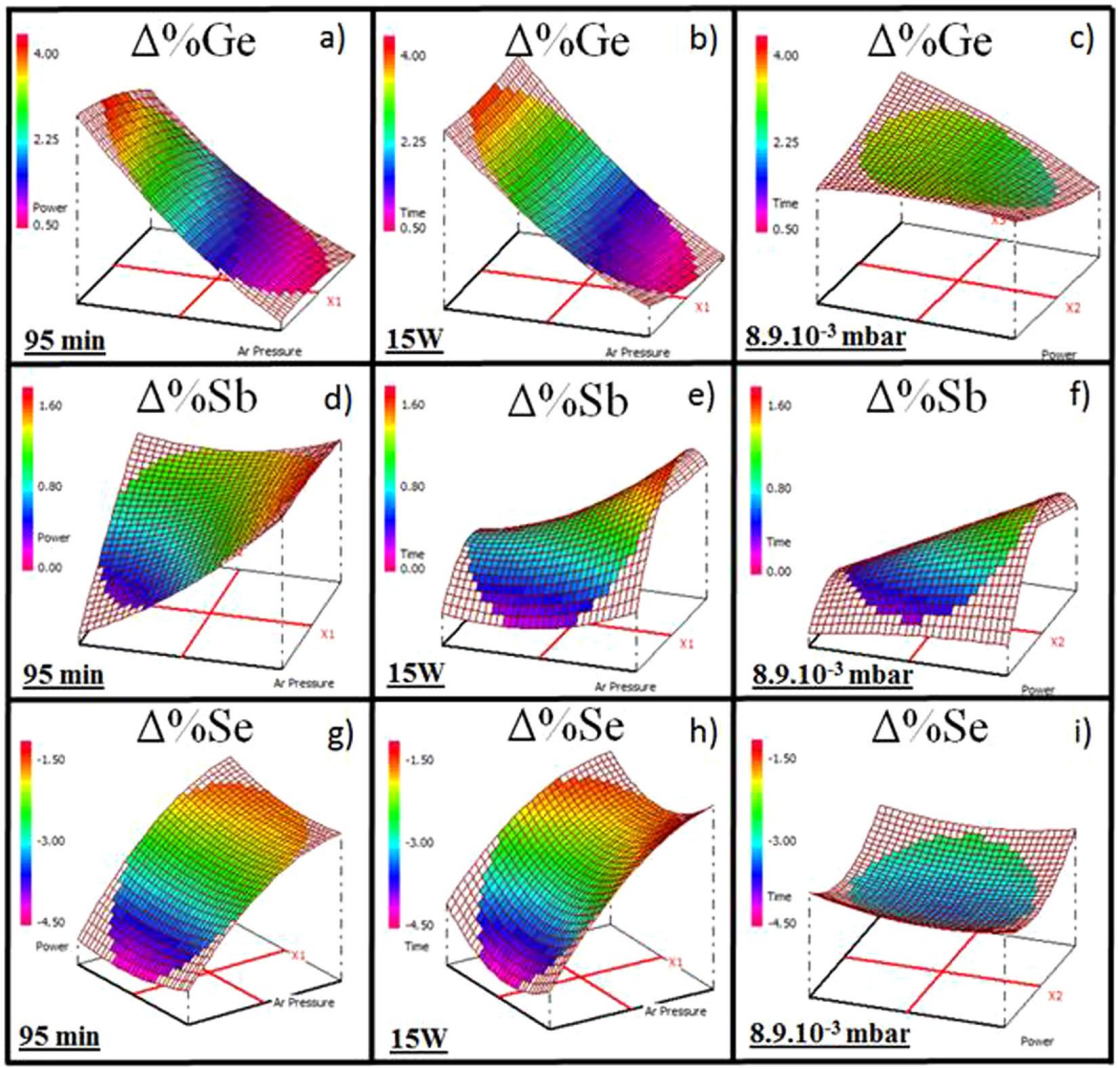
Variation of the chemical composition of the Ge_12.5_Sb_25_Se_62.5_ thin films for a fixed deposition time 95 min (**a**) Δ%Ge, (**d**) Δ%Sb, (**g**) Δ%Se, for a fixed working power 15 W (**b**) Δ%Ge, (**e**) Δ%Sb, (**h**) Δ%Se, for a fixed Ar pressure 8.9.10^−3^ mbar (**c**) Δ%Ge, (**f**) Δ%Sb, (**i**) Δ%Se.

The variation of the chemical composition of the Ge_12.5_Sb_25_Se_62.5_ thin films is presented in Fig. 2. The thin films show a systematic over-stoichiometric content of germanium (ΔGe varies from 0.7 to 3.5%, average 1.9%, deviation 0.1%). The response decreases when the Ar pressure increases (Fig. 2a and b) while the deposition time and the working power have no effect (Fig. 2c). Similarly, antimony is slightly in excess (ΔSb varies from 1.4 to 0%, average 0.8%, deviation 0.3%). For a long deposition time, the variation of the antimony composition is less negligible: from 0.4 to 1.2% for a fixed working power (15 W, Fig. 2e) varying with change in pressure and from 0.2 to 0.7% for a fixed Ar pressure (8.9.10^−3^ mbar, Fig. 2f). We can also observe a deficiency in selenium element (ΔSe varies from −4.5 to −1.7%, average −2.6%, deviation 0.2%) which is more pronounced in the case of a low Ar pressure. Regardless of the deposition time and the working power, for the fixed Ar pressure 8.9.10^−3^ mbar, an almost perfect uniformity of the response surface is observed with constant ΔSe = −3.5% (Fig. 2i).

The difference between composition of the target and the thin films can be explained to a certain extent by a sputtering yield of each component of the complex Ge-Sb-Se targets. The sputtering yield is defined as the average number of ejected atoms from the surface of the target per incident Ar^+^ ion. In the case of a single component target, the sputtering yield is influenced by different factors - mainly by the energy and the incident angle of the Ar ions, the relative masses of the incident ions and target atoms, and the surface binding energy of the target atoms^43–45^. In the literature, predictive semi-empirical approaches have been proposed for monoatomic elemental solids to enable calculation of the sputtering yield based on the theory of Sigmund and sputtering yields were found to vary periodically with the element’s atomic number^46–50^. For comparison purposes, the sputtering yield values of single component targets around 1–1.5, 2.8–4.1 and 5.2–7.4 atom/ion for germanium, antimony and selenium targets, respectively, can be mentioned. These sputtering yields data represent the number of atoms ejected from the target per argon ion striking normally on the surface of the target with a classical kinetic energy range from 500 to 1000 eV. Nevertheless, magnetron design factors such as magnetic field strength, radio-frequency (13.56 MHz) and process parameters will affect the sputtering yields.

Furthermore, the sputtering deposition of a multicomponent target is even more complex and perplexing than that one of the single component targets. For Ge-Sb-Se amorphous targets, other factors have to be considered such as the chemical bond energy of Ge-Se and Sb-Se bonds which predominate in the glass network (around 235–207 kJ/mol and ~217–184 kJ/mol depending on data source^21, 51–53^), atomic weights of the three elements constituting the target, amorphous nature, density affecting a surface binding energy of the target atoms. It can be indicated that chemical bonds of many compounds are stronger than those of the pure elements leading to lower sputtering yields for complex targets^54^. Considering the higher sputtering yield of a chalcogen element in the single component target, it can be assumed that the Ge-Sb-Se target surface will quickly impoverish in Se, until an equilibrium composition is reached leading to deficiency in selenium at the surface of the target. Conversely, the surface layer of the target will be enriched with the element having the lower sputtering yield, i.e. germanium. However, it must be remembered that the target is not composed of a single element but from three elements and mainly due to their difference in size, density and binding energies, the performance of sputtering of each elements will be affected. Ge-Sb-Se amorphous targets are composed of elements with different atomic weights (A_r_(Ge), A_r_(Se) < A_r_(Sb)) and relatively similar chemical bond energies even if it can be noticed that Ge-Se bonds present higher values than Sb-Se bonds. Considering the Ge-Se and Sb-Se bo﻿nd energy, the difference between the sputtering yield of antimony and germanium observed for pure targets should be respected for the multicomponent target, i.e Y_SP_(Ge) < Y_SP_(Sb). Selenium which is lighter than antimony and rather close to germanium weight will tend to be ejected more easily. Usually during sputtering of a multicomponent target, the more volatile element is preferentially eliminated (i.e., selenium from Ge-Sb-Se target), so the surface of the sputtering target is enriched by less volatile constituents^54^. That can amplify the higher sputtering yield of selenium compared to Ge and Sb in the Ge-Sb-Se multicomponent target. Indeed, the sputtering yield trend of single component targets Y_SP_(Ge) < Y_SP_(Sb) < Y_SP_(Se) is expected to be maintained in the Ge-Sb-Se targets.

The sputtering yields will be also more or less modified by deposition parameters (Ar pressure, working power, deposition time) and for each set of deposition parameters, different sputtering yields for each atom will lead to a change in the initial composition of the Ge-Sb-Se target. This change is counterbalanced by the fact that the concentration of elements preferably sputtered on the surface will decrease to achieve a so-called equilibrium composition at the surface of the target. Typically, the equilibrium composition is achieved after deposition of the first ten nanometers of the target. As we have discussed previously, the equilibrium composition can drift from the stoichiometric one if the sputtering yields are quite different from each other. This behavior can lead to a loss of stoichiometry in the deposited film from the target material. The sputtering yield for elements conventionally present in the chalcogenide layers has been reported in the literature with a high yield for chalcogens (selenium, then tellurium and finally sulfur)^43^. In many cases, some of the lightest and most volatile species, such as chalcogens, are lost in the transfer between the target and the substrate or the probability of reaction with the more condensable species on the substrate surface is less effective^54^. Thus, it is reasonable to expect a loss of selenium for the sputtered thin film compared to the target stoichiometry.

Growth and characteristics of the thin films can be better understood knowing the nature and kinetic properties of the sputtered particles. At incident ion energy of a few hundred of electron volts, most of the sputtered atoms of single component or alloy targets are composed of neutral single atoms; only partially ionized or forming clusters (only few %)^45^. Energy distribution of the sputtered atoms leaving the target can be expressed using the model developed by Thompson^55^. Such distribution mainly depends on the global characteristics of the sputtered material (reduced mass and cohesive energy) and sputtering ion energy. In the present case, sputtering is carried out at a low power and in all our pressure condition, all sputtered atoms are assumed to have a similar energy distribution leaving the target^11^. Energy distribution of the sputtered atoms reaching the substrate may be greatly modified by average number of collisions during their transfer to the substrate. We have estimated that the average atom energy is typically 2–4 eV, both at a target and substrate position, for pressure from 5.10^−3^ up to 1.10^−2^ mbar, while a much lower value of ~0.1 eV is expected for higher pressure. Thus, the chemical composition of films deposited at a lower pressure mainly follows the sputtering yields of Se, Sb and Ge; the particles with a long mean free path are not so much perturbed by collisions during the transfer from the target to the substrate. It is not the case of the deposition at higher pressure (>7.5.10^−3^ mbar) where the sputtered particles are more affected by multiple collisions and thermalization of the particles which will modify the composition, structure and morphology of the thin films.

Finally, it can be observed that the average change of the film composition compared to target depending on the deposition parameters follows the sputtering yields trend for the two Ge_28.1_Sb_6.3_Se_65.6_ and Ge_12.5_Sb_25_Se_62.5_ targets: deficiency in selenium (ΔSe_mean_: −1.3% and −2.6%), excess of germanium (ΔGe_mean_: 1% and 1.9%) and relative stability of antimony composition (ΔSb_mean_: 0.3% and 0.8%). The composition of the films from the two targets ranges around these average values, which was more pronounced in the case of the Ge_28.1_Sb_6.3_Se_65.6_ target and Ge and Se elements. These composition variations depend on the choice of deposition parameters which can affect the sputtering yield of the three elements and also their transfer and condensation on the substrate. Effectively, we noticed that for some specific deposition parameters set, it is possible to be closer to stoichiometry of the target mainly obtained with a pressure higher than 1.10^−2^ mbar for which the influence of transfer and condensation of sputtered atoms will be important.

Lastly, the knowledge of the response surfaces depending on the deposition parameters set from the experimental design analysis enables to control the film composition. Elaboration of an optical waveguide requires a structure composed with thick layers (1–5 µm) and alternate compositions (Ge_28.1_Sb_6.3_Se_65.6_ and Ge_12.5_Sb_25_Se_62.5_). This study reveals a deposition parameters set allowing obtaining a good homogeneity of composition especially for a long deposition time which means that a layers component structure of the optical waveguide will have a good homogeneity even for thicker layers.

### Refractive indices in near-IR and mid-IR

**Figure 3:**
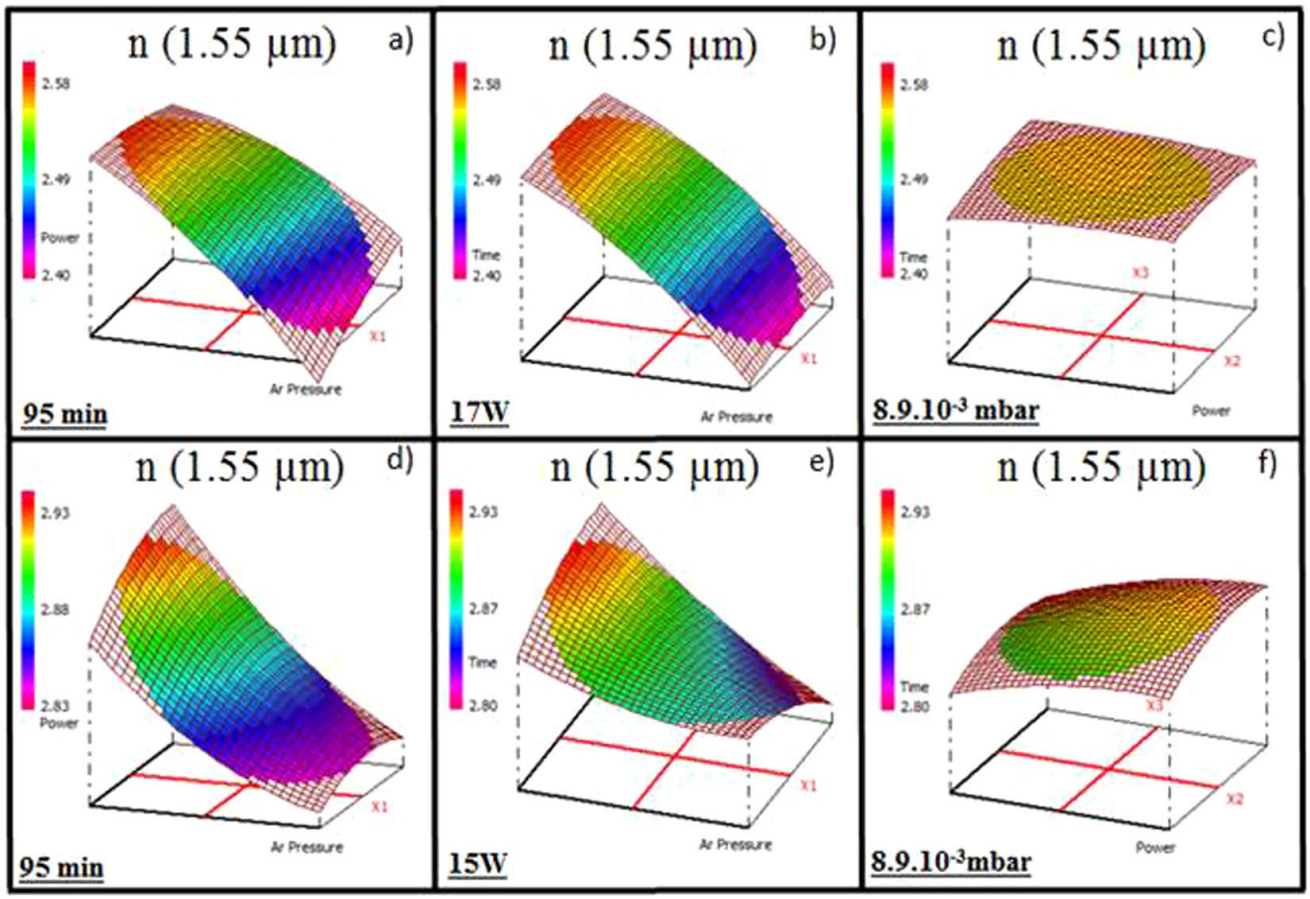
Variation of the refractive index (1.55 µm) of the Ge_28.1_Sb_6.3_Se_65.6_ thin films for (**a**) a fixed deposition time of 95 min, (**b**) a fixed working power of 17 W, (**c**) a fixed Ar pressure of 8.9.10^−3^ mbar and the Ge_12.5_Sb_25_Se_62.5_ thin films for (**d**) a fixed deposition time 95 min, (**e**) a fixed working power 15 W, (**f**) a fixed Ar pressure 8.9.10^−3^ mbar.

**Figure 4:**
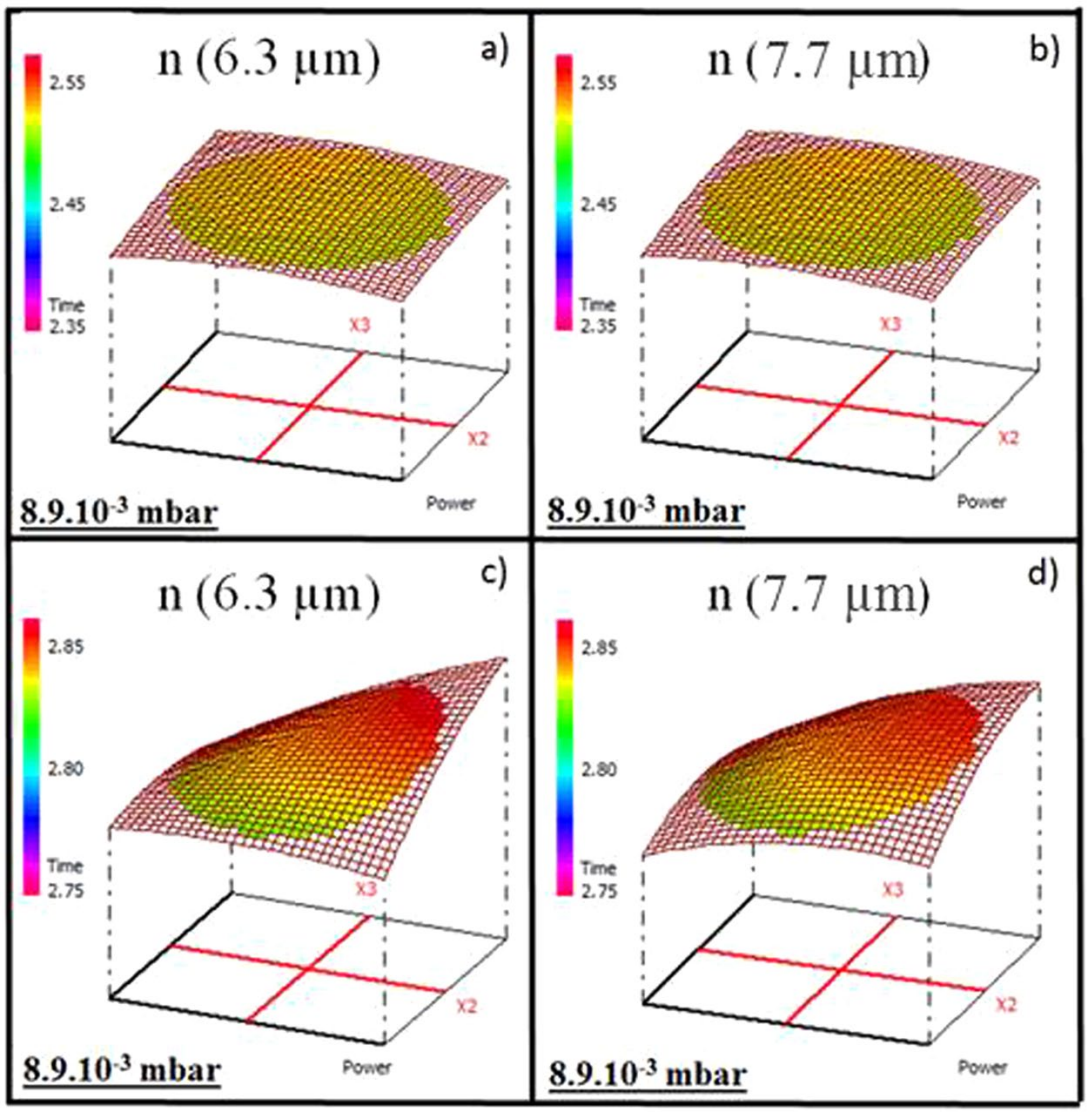
Variation of the refractive index of the thin films for a fixed Ar pressure (**a**) Ge_28.1_Sb_6.3_Se_65.6_, λ = 6.3 µm, (**b**) Ge_28.1_Sb_6.3_Se_65.6_, λ = 7.7 µm, (**c**) Ge_12.5_Sb_25_Se_62.5_, λ = 6.3 µm, (**d**) Ge_12.5_Sb_25_Se_62.5_, λ = 7.7 µm.

The knowledge and control of the refractive index contrast between the two chalcogenide layers are essential to obtain an optical waveguide operating in the mid-IR^10, 11, 39^. Using the Cody-Lorentz model, refractive indices in near-IR and mid-IR (±0.01) were extracted from variable angle spectroscopic ellipsometry (VASE) data. The variations of the refractive indices of the Ge_28.1_Sb_6.3_Se_65.6_ and Ge_12.5_Sb_25_Se_62.5_ thin films in near-IR (1.55 µm) are presented in Fig. 3: for a fixed deposition time of 95 min (a and d), for a fixed working power of 17 W (b) and 15 W (e) and for a fixed Ar pressure of about 8.9.10^−3^ mbar (c and f). Figure 4 presents the variation of the refractive indices of the Ge_28.1_Sb_6.3_Se_65.6_ and Ge_12.5_Sb_25_Se_62.5_ thin films in mid-IR for a fixed Ar pressure 8.9.10^−3^ mbar at 6.3 µm (a and c) and at 7.7 µm (b and d). The refractive index in near-IR (λ = 1.55 µm) varies from 2.41 to 2.56 and from 2.83 to 2.93 for the Ge_28.1_Sb_6.3_Se_65.6_ and Ge_12.5_Sb_25_Se_62.5_ thin films, respectively. The Ar pressure has a predominant influence on the refractive index and a large decrease of the response is observed when the Ar pressure increases. The deposition time and the working power have almost no effect on the refractive index (Fig. 3c and f). Indeed, when the Ar pressure is fixed at 8.9.10^−3^ mbar, variation of the refractive index considering the uncertainty of ellipsometry (0.01) and experimental variance is weak, almost unnoticeable for the Ge_12.5_Sb_25_Se_62.5_ thin films (n (1.55 µm) = 2.89–2.88) and there is no variation for the Ge_28.1_Sb_6.3_Se_65.6_ thin films (n (1.55 µm) = 2.53). This behavior is confirmed by the results in mid-IR following a classical chromatic dispersion. The refractive index in mid-IR (6.3 µm), for a fixed Ar pressure 8.9.10^−3^ mbar and for the two compositions (Ge_28.1_Sb_6.3_Se_65.6_ and Ge_12.5_Sb_25_Se_62.5_), has a very weak variation (0.01), as shown in Fig. 4. Results in near-IR (1.55 µm) and in mid-IR (6.3 µm and 7.7 µm) are correlated; refractive indices have the same behavior whatever the wavelength is. Linearity of the response is observed between the refractive index in near-IR and mid-IR. The variation of the refractive index of as–deposited films is usually mostly driven by a chemical composition change and/or a change in the morphology of the thin film resulting from the Ar pressure change^35^. For the Ge_28.1_Sb_6.3_Se_65.6_ thin films, the lowest refractive indices are observed for a deficiency in Ge and an excess in Se. For the Ge_12.5_Sb_25_Se_62.5_ thin films, when the deficiency in selenium element is lower, the refractive indices are also lowest. Considering Ge-Sb-Se bulk glasses, the refractive index and density are decreasing when germanium content is increasing for a fixed antimony concentration and an over-stoichiometric selenium concentration. The decrease in the refractive index cannot be explained by a change in the composition. It can be underlined that the Ar pressure is the most predominant factor for both compositions and all over the range of wavelengths considered (in near and mid-IR).

### Band-gap energy

**Figure 5:**
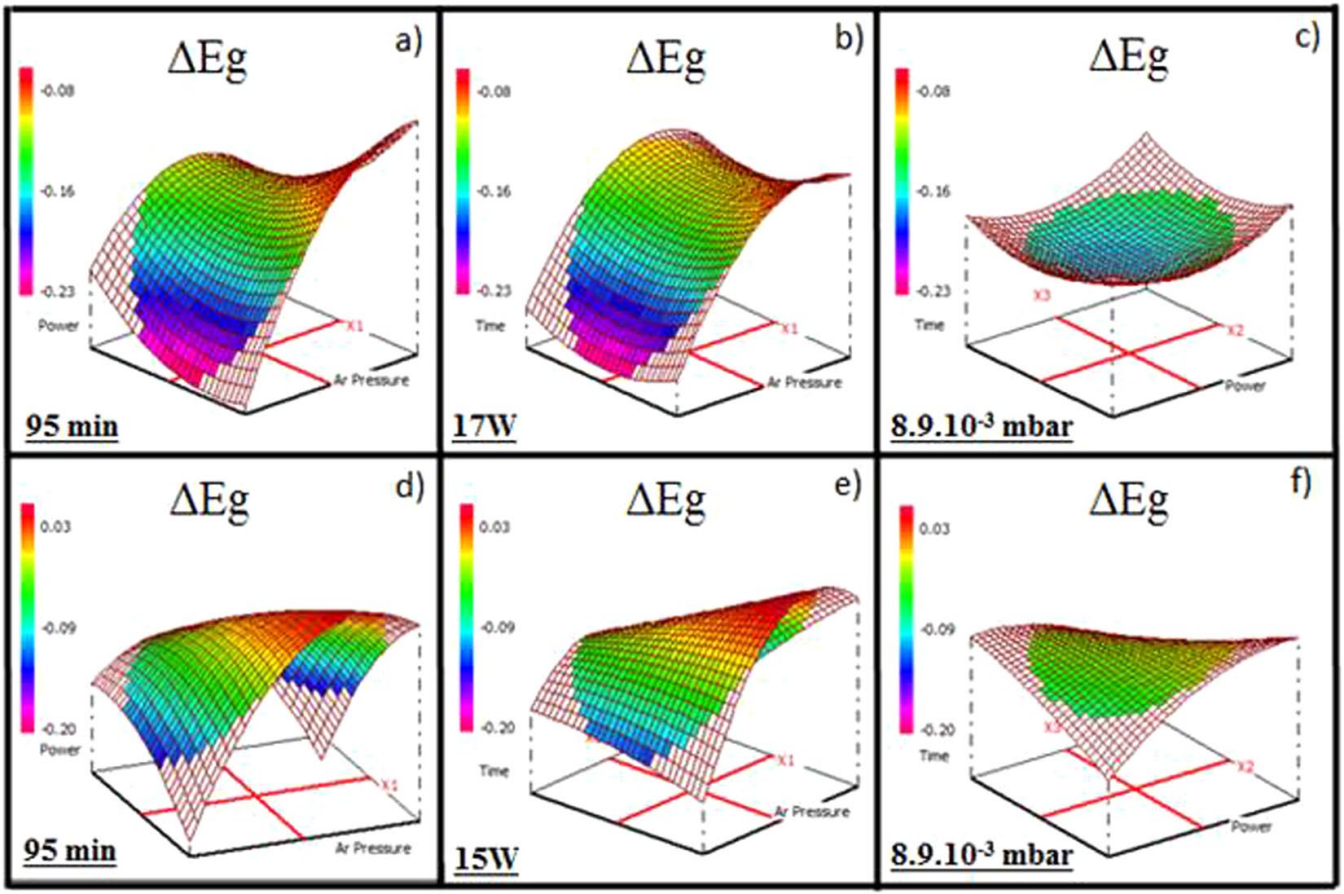
Variation of ΔE_g_ of the Ge_28.1_Sb_6.3_Se_65.6_ thin films for (**a**) a fixed deposition time of 95 min, (**b**) a fixed working power of 17 W, (**c**) a fixed Ar pressure of 8.9.10^−3^ mbar and the Ge_12.5_Sb_25_Se_62.5_thin films for (**d**) a fixed deposition time of 95 min, (**e**) a fixed working power of 15 W, (**f**) a fixed Ar pressure of 8.9.10^−3^ mbar.

To determine the influence of deposition parameters on the band-gap energy, ΔE_g_ was chosen as${{\rm{\Delta }}{\rm{E}}}_{{\rm{g}}}={{\rm{E}}}_{{\rm{g}}}({\rm{thin}}\,{\rm{film}})-{{\rm{E}}}_{{\rm{g}}}({\rm{target}})$. The band-gap energy (±0.01 eV) values of the Ge_28.1_Sb_6.3_Se_65.6_ and Ge_12.5_Sb_25_Se_62.5_ targets were extracted from VASE data as 2.01 and 1.62 eV. The variations of ΔE_g_ for the Ge_28.1_Sb_6.3_Se_65.6_ and Ge_12.5_Sb_25_Se_62.5_ thin films based on VASE data are presented in Fig. 5 for a fixed deposition time (95 min, a and d), a fixed working power 17 W (Ge_28.1_Sb_6.3_Se_65.6_) and 15 W (Ge_12.5_Sb_25_Se_62.5_) (b and e) and for a fixed Ar pressure 8.9.10^−3^ mbar (c and f).

Concerning the Ge_28.1_Sb_6.3_Se_65.6_ thin films, ΔE_g_ varies from −0.23 to −0.08 eV (average: −0.14 eV; deviation: 0.01 eV). This means that the band-gap energy of the thin films is systematically lower than that one of the target. The Ar pressure is the preponderant factor while the deposition time and the working power have a small influence on the response. In fact, for a fixed deposition time of 95 min, ΔE_g_ varies from −0.23 eV to −0.08 eV; for a fixed working power 17 W, ΔEg varies from −0.22 eV to −0.13 eV while for a fixed Ar pressure 8.9.10^−3^ mbar, the variation of ΔE_g_ is much weaker, from −0.15 eV to −0.17 eV. An increase of the Ar pressure (from 5.10^−3^ mbar to 5.10^−2^ mbar) leads to a decrease of ΔE_g_ variation (from −0.23 eV to −0.13 eV).

The results for the Ge_12.5_Sb_25_Se_62.5_ thin films are different; a clear influence of each factor (Ar pressure, working power and deposition time) is observed. ΔE_g_ varies from −0.15 to 0.03 eV (average: −0.05 eV, deviation: 0.01 eV). Therefore, the band-gap energy of the Ge_12.5_Sb_25_Se_62.5_ thin films can be lower or slightly higher than the band-gap energy of the target. A decrease of the response is noted for different factors set combining a high Ar pressure to a high working power (deposition time 95 min, ΔE_g_ = −0.12 eV), a high Ar pressure to a long deposition time (working power 15 W, ΔE_g_ = −0.12 eV) or a low Ar pressure associated to a low working power (deposition time 95 min, ΔE_g_ = −0.14 eV).

Variation of the band-gap energy is mainly due to the variation of the chemical composition, such as Se deficiency and Sb excess, the variation of a glass network structure, the variation of defects affected by deposition parameters^38^. All these variation will generate a modification of the electronic band structure: a change in the contribution of Se lonely electron pairs, of bonding electrons involved in homopolar bonds and of Ge-Se, Sb-Se bonds forming the top of the valence band. The influence of deposition parameters on E_g_ differs according to the target. Globally, the Ge_28.1_Sb_6.3_Se_65.6_ thin films tend to have smaller E_g_ than the target which can be mainly explained by the deficiency of selenium in case of higher ΔE_g_. Whereas the Ge_12.5_Sb_25_Se_62.5_ thin films tend to have stable E_g_ close to the target especially for a low working power even if a Se deficit of 2–3% is observed. The different behavior of the two targets is likely related to the Ge/Sb ratio difference influencing the electronic structure of the amorphous network. The systematic negative values of ΔE_g_ of Ge_28.1_Sb_6.3_Se_65.6_ whatever the composition or the ΔE_g_ ~ 0 eV of Ge_12.5_Sb_25_Se_62.5_ even if a Se deficit of 2–3% have probably origin in the glass network structure variation and defects formation compare to the bulk glass target. Further study will be performed to better understand the band-gap variation according to the deposition parameters.

### Deposition rate

**Figure 6:**
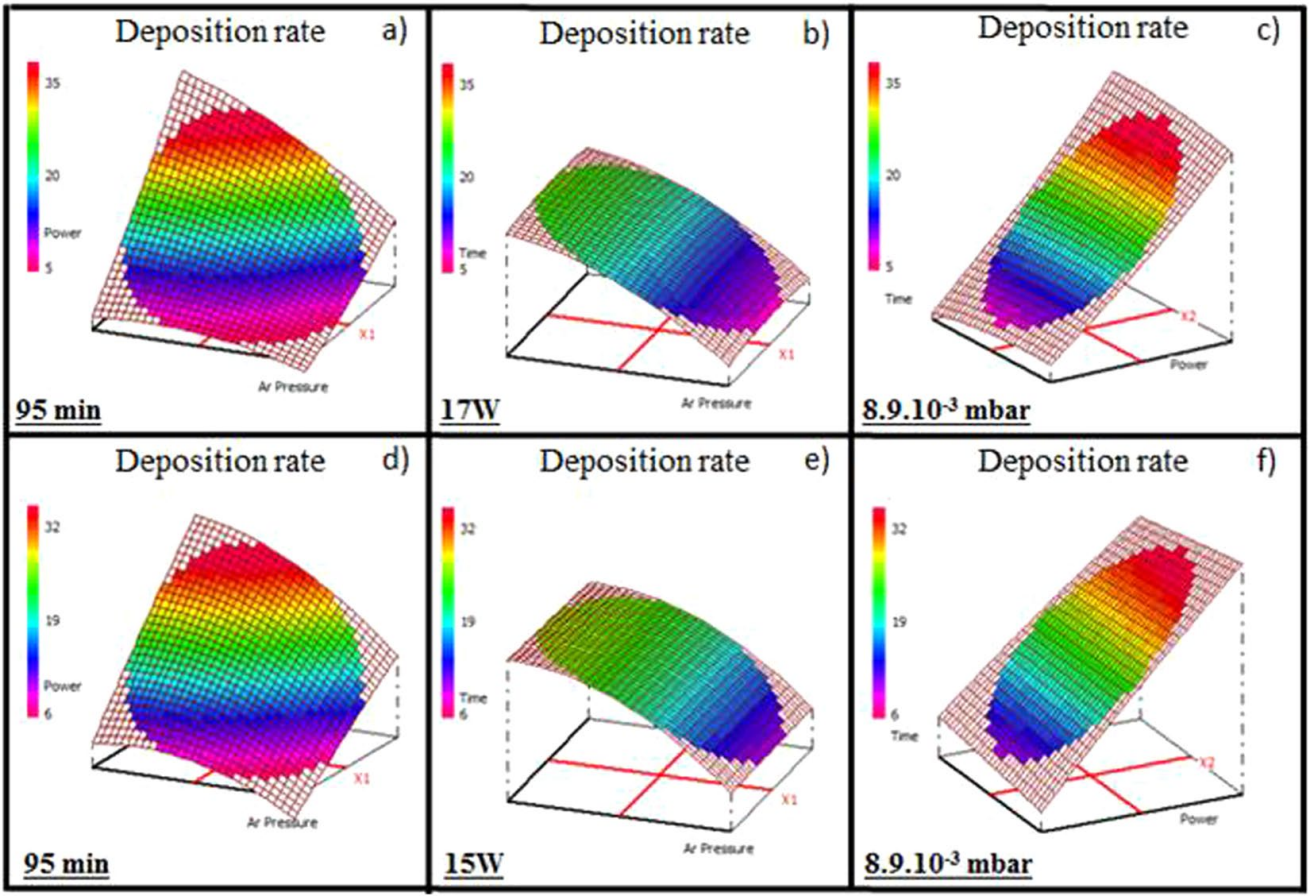
Variation of the deposition rate of the Ge_28.1_Sb_6.3_Se_65.6_ thin films for (**a**) a fixed deposition time of 95 min, (**b**) a fixed working power of 17 W, (**c**) a fixed Ar pressure of 8.9.10^−3^ mbar and the Ge_12.5_Sb_25_Se_62.5_ thin films for (**d**) a fixed deposition time of 95 min, (**e**) a fixed working power of 15 W, (**f**) a fixed Ar pressure of 8.9.10^−3^ mbar.

Thin films thicknesses determined from VASE data (±1 nm) were used to determine deposition rates. The deposition rates range from 5 to 38 nm.min^−1^ and from 7 to 33 nm.min^−1^ for Ge_28.1_Sb_6.3_Se_65.6_ and Ge_12.5_Sb_25_Se_62.5_, respectively (Tables 3 and 4). The results are the same for both compositions: an Ar pressure and a working power are the two influential factors while deposition time has no effect on the response (Fig. 6). An increase of the deposition rate is observed for a low Ar pressure and a high working power. In the classical sputtering regime, the sputtering yield increases linearly with an incident ion energy and in many cases, the deposition rate goes up with the working power^43^. The deposition rate is usually decreased by a higher pressure if the sputtered particles will undergo multiple collisions and thermalization during the transfer to the substrate^45^. For a fixed deposition time (95 min), the deposition rates vary from 5 to 38 nm.min^−1^ and from 7 to 33 nm.min^−1^ for the Ge_28.1_Sb_6.3_Se_65.6_ (Fig. 6a) and Ge_12.5_Sb_25_Se_62.5_ (Fig. 6d) thin films, respectively. When a working power is fixed (17 W), the deposition rates vary from 8 to 23 nm.min^−1^ for the Ge_28.1_Sb_6.3_Se_65.6_ thin films (Fig. 6b) and from 12 to 21 nm.min^−1^ for the Ge_12.5_Sb_25_Se_62.5_ thin films (Fig. 6e). At Ar pressure of 8.9.10^−3^ mbar, the deposition rates vary from 8 to 35 nm.min^−1^ for the Ge_28.1_Sb_6.3_Se_65.6_ thin films (Fig. 6c) and from 12 to 33 nm.min^−1^ for the Ge_12.5_Sb_25_Se_62.5_ thin films (Fig. 6f). It can be concluded that a working power is the most influential factor; the variation of this deposition parameter induces a large variation of the deposition rate. For a long deposition experiment, there is no variation of the deposition rate; therefore thicknesses of thick layers (1–5 µm) for an optical structure can be easily controlled.

### AFM Surface roughness

**Figure 7:**
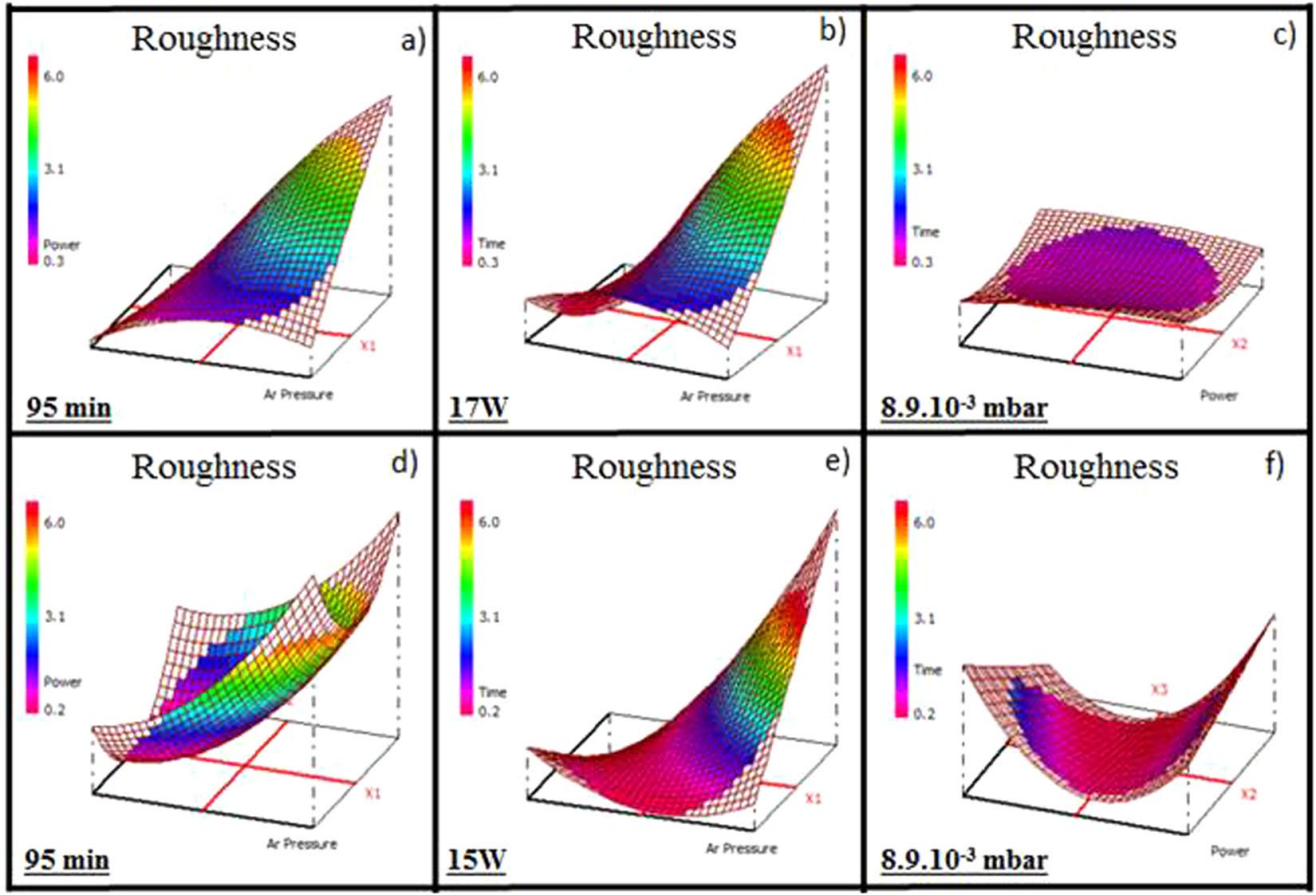
Variation of the surface roughness of the Ge_28.1_Sb_6.3_Se_65.6_ thin films for (**a**) a fixed deposition time of 95 min, (**b**) a fixed working power of 17 W, (**c**) a fixed Ar pressure of 8.9.10^−3^ mbar and the Ge_12.5_Sb_25_Se_62.__5_ thin films for (**d**) a fixed deposition time of 95 min, (**e**) a fixed working power of 15 W, (**f**) a fixed Ar pressure of 8.9.10^−3^ mbar.

The surface roughness of the Ge_28.1_Sb_6.3_Se_65.6_ thin films varies from 0.37 to 6.46 nm (Table 3). Figure 7a presents the variation of the surface roughness for a fixed deposition time of 95 min (0.4 to 4.6 nm); the response decreases when the Ar pressure decreases and the working power has no clear effect. For a fixed working power of 17 W (Fig. 7b), the surface roughness varies from 0.4 to 5.2 nm; as the working power, the deposition time has almost no effect dissimilar to the Ar pressure. Finally, for a fixed Ar pressure of 8.9.10^−3^ mbar (Fig. 7c), the surface roughness varies from 1.1 to 1.5 nm and the response decreases slightly for a short deposition time and a low working power. It can be concluded that the Ar pressure has a predominant influence. Especially in the domain of the low Ar pressure, the working power and the deposition time have no effect on the surface roughness. The surface roughness of the Ge_12.5_Sb_25_Se_62.5_ thin films (Table 4) has a similar comportment to the Ge_28.1_Sb_6.3_Se_65.6_ thin films; it ranges from 0.45 to 6.26 nm. For a fixed deposition time of 95 min (Fig. 7d), the surface roughness varies from 0.45 to 5.2 nm and the response decreases for a low Ar pressure and for an intermediate working power. For a fixed working power 15 W (Fig. 7e), the surface roughness varies from 0.45 to 5.7 nm; the response is lower for a low Ar pressure and the deposition time has no effect for a low Ar pressure. In the same way, for a fixed Ar pressure 8.9.10^−3^ mbar (Fig. 7f), the surface roughness varies from 0.5 to 2.0 nm and the lower values (0.5 nm) are obtained for an intermediate working power. To conclude, the Ar pressure is the most influential factor and allows obtaining a wide range of the surface roughness. For a low Ar pressure (<1.6.10^−2^ mbar), the working power and the deposition time have a small influence. However, the surface roughness slightly decreases for a short deposition time and for an intermediate working power.

In order to reduce optical losses in the optical waveguides, the thin films with a low surface roughness are required. The deposition parameter that seems to be the most suitable is a lower Ar pressure (≤1.6.10^−2^ mbar). The working power has also a small influence on the surface roughness, especially for a low Ar pressure, an appropriate working power is intermediate for Ge_12.5_Sb_25_Se_62.5_.

## Methods

The glass targets for RF magnetron sputtering of the thin films (GeSe_2_)_100−x_(Sb_2_Se_3_)_x_ with the nominal composition of Ge_28.1_Sb_6.3_Se_65.6_ (x = 10) and Ge_12.5_Sb_25_Se_62.5_ (x = 50) were prepared using the conventional method of melting and quenching from high-purity elements (5 N). Selenium had been previously purified by a static distillation. The elements were weighted in an appropriate amount, inserted in a specific silica glass ampoule under vacuum and then sealed. The elements had been melted in a rocking furnace at 850 °C and then the ampoules with the melt were quenched. The resulting glasses were annealed at a temperature close to their glass transition temperature (Tg): at ~330 and ~205 °C for Ge_28.1_Sb_6.3_Se_65.6_ and Ge_12.5_Sb_25_Se_62.5_, respectively. Chalcogenide glass discs with a diameter of 50 mm and a thickness of 3.5 mm were obtained after cutting of the glass rods followed by polishing. The glass discs were used as sputtering targets for a thin films deposition. The chalcogenide thin films were deposited by RF magnetron sputtering on silicon substrates. The deposition was carried out at a working Ar pressure in a range of 5.10^−2^–5.10^−3^ mbar. The sputtering was maintained at a low RF working power (10–25 W) considering the insulator character of the targets and the requirement of amorphous layers fabrication. An off-axis substrates rotation was operated during the deposition process. The silicon substrates were positioned at the target-to-substrate distance of 5 cm.

### Thin films characterization

The chemical composition of the Ge_28.1_Sb_6.3_Se_65.6_ and Ge_12.5_Sb_25_Se_62.5_ thin films were measured using a scanning electron microscope with an EDS (JSM 6400-OXFORD Link INCA). Linear refractive indices spectral dependencies of the thin films, thicknesses, consequently deposition rates, as well as optical band-gap values (*E*_*g*_), were obtained from the analysis of VASE data measured using two ellipsometers^56^: a rotating analyzer ellipsometer measuring in UV-Vis-NIR (300–2300 nm) and a rotating compensator ellipsometer working in mid-IR (~1.7–30 µm) (both J. A. Woollam Co., Inc., Lincoln, NE, USA). The VASE measurements parameters are as follows: angles of incidence of 65°, 70° and 75°, resolution of UV-Vis-NIR ellipsometer of 20, 10 and 5 nm, resolution of the mid-IR ellipsometer of 2, 8 and 16 cm^−1^. The used resolution was selected in accordance with the estimated thickness of the thin films. The Cody-Lorentz model^57, 58^ was used to analyze VASE data. This model is appropriate for the description of amorphous chalcogenide optical functions^59^. The chalcogenide thin films roughness was studied by an atomic-force microscopy (AFM, Ntegra Prima, NT-MDT). The tapping mode imaging was used on an area of 1 µm × 1 µm.

## Conclusions

In summary, Ar pressure, working power and deposition time were selected as potentially the most influential factors for the chalcogenide thin film deposition in the experimental design approach. The results of the experimental design analysis confirm the great influence of the Ar pressure. It appears clearly that all responses studied here are influenced by the Ar pressure: the chemical composition, the refractive index in near-IR (1.55 µm) and mid-IR (6.3 µm and 7.7 µm), the band-gap energy, the deposition rate and the surface roughness. Globally, the composition presents some deficit in selenium and an excess of germanium while antimony content is more stable, in good agreement with their sputtering yields. The thin films tend to better reflect the chemical composition of the target for a high Ar pressure. The band-gap energy and the surface roughness are also mainly affected by the Ar pressure while the deposition time and the working power have almost no effects. The deposition rate is strongly changed by the working power (increasing with the power), and to some extend by the high Ar pressure which is reducing it. Moreover, refractive indices are highly influenced by the Ar pressure; a significant decrease is observed for an increase of the pressure related to an important change of the morphology/porosity and the roughness of the films.

Depending on the intended applications and therefore desired thin films characteristics, mappings of the experimental design help to select suitable deposition parameters. To elaborate an optical waveguide composed of buffer and core selenide layers for sensor applications^10, 38, 39^: a stable composition during deposition not so far from a target composition, a low surface roughness, a constancy of the refractive index contrast between the two layers and a high deposition rate for the buffer layer are desired. To better respond to these characteristics, it is necessary to work with the most suitable Ar pressure. Working with an intermediate Ar pressure of about 1.10^−2^ mbar seems to be a good compromise maintaining an adequate stable composition, a low roughness, no porosity and a suitable refractive index contrast between the two targets. A power of 20 W is preferred for the Ge_28.1_Sb_6.3_Se_65.6_ target, serving as the buffer layer due to a lower refractive index, with the deposition rate of 33 nm/min allowing deposition of a thick layer. For the Ge_12.5_Sb_25_Se_62.5_ thinner layer, a lower power of about 10 W is preferred due to necessity to stabilize the refractive index and the composition during the deposition process with a lower deposition rate of about 11 nm/min and a quite low roughness required for a core layer. Furthermore, to complete this study, a structural analysis by Raman spectroscopy and XPS was performed to investigate the influence of the Ar pressure on structural features of the sputtered amorphous films^38^. However, further investigation will be performed in order to better understand the behavior of the band-gap of the Ge-Sb-Se sputtered films.
